# Genes controlling vaccine responses and disease resistance to respiratory viral pathogens in cattle

**DOI:** 10.1016/j.vetimm.2011.05.009

**Published:** 2012-07-15

**Authors:** Elizabeth J. Glass, Rebecca Baxter, Richard J. Leach, Oliver C. Jann

**Affiliations:** The Roslin Institute and Royal (Dick) School of Veterinary Studies, University of Edinburgh, Easter Bush, Midlothian, EH25 9RG, UK

**Keywords:** Cattle, Genetics, Vaccine response, Bovine respiratory syncytial virus, Foot and mouth disease virus, Quantitative trait loci, Whole genome scan, Polymorphism, Bovine major histocompatibility complex, BoLA, Toll like receptor

## Abstract

Farm animals remain at risk of endemic, exotic and newly emerging viruses. Vaccination is often promoted as the best possible solution, and yet for many pathogens, either there are no appropriate vaccines or those that are available are far from ideal. A complementary approach to disease control may be to identify genes and chromosomal regions that underlie genetic variation in disease resistance and response to vaccination. However, identification of the causal polymorphisms is not straightforward as it generally requires large numbers of animals with linked phenotypes and genotypes. Investigation of genes underlying complex traits such as resistance or response to viral pathogens requires several genetic approaches including candidate genes deduced from knowledge about the cellular pathways leading to protection or pathology, or unbiased whole genome scans using markers spread across the genome.

Evidence for host genetic variation exists for a number of viral diseases in cattle including bovine respiratory disease and anecdotally, foot and mouth disease virus (FMDV). We immunised and vaccinated a cattle cross herd with a 40-mer peptide derived from FMDV and a vaccine against bovine respiratory syncytial virus (BRSV). Genetic variation has been quantified. A candidate gene approach has grouped high and low antibody and T cell responders by common motifs in the peptide binding pockets of the bovine major histocompatibility complex (BoLA) DRB3 gene. This suggests that vaccines with a minimal number of epitopes that are recognised by most cattle could be designed. Whole genome scans using microsatellite and single nucleotide polymorphism (SNP) markers has revealed many novel quantitative trait loci (QTL) and SNP markers controlling both humoral and cell-mediated immunity, some of which are in genes of known immunological relevance including the toll-like receptors (TLRs).

The sequencing, assembly and annotation of livestock genomes and is continuing apace. In addition, provision of high-density SNP chips should make it possible to link phenotypes with genotypes in field populations without the need for structured populations or pedigree information. This will hopefully enable fine mapping of QTL and ultimate identification of the causal gene(s). The research could lead to selection of animals that are more resistant to disease and new ways to improve vaccine efficacy.

## 1 Genetic variation in disease resistance and vaccine response

Endemic and exotic pathogens continue to have a significant effect on livestock with high economic costs and welfare implications worldwide. In addition emerging pathogens are increasingly recognised as a threat to biosecurity and zoonotic pathogens and chemical usage still cause food safety hazards. These concerns remain despite increasing regulation and control measures such as good management and the availability of vaccines at least to some pathogens. Globalisation of trade and climate change bring new urgency to find ways to reduce the impact of infectious disease which remains a barrier to further improvement in livestock efficiency in both the developed and developing world.

One potential solution is to breed livestock that are inherently more resistant or tolerant to prevailing pathogens. However it is not easy to identify markers and genes that would enable selection of such desirable traits, partly because of the cost and logistics of collecting appropriate large-scale data either from the field or experimental challenges. Thus there is a phenotype gap. Furthermore resistance or tolerance traits are likely to be underpinned by many genes of small effect, making it more difficult to identify them. Another solution would be to design more effective vaccines. After the foot and mouth disease virus (FMDV) outbreak in the U.K. in 2001, the Royal Society stated that research should concentrate on developing an ideal vaccine that would be “safe, synthetic, induce sterile immunity, in one shot, be cost effective and protect all animals” (Royal Society, 2002). It is debatable whether any livestock vaccine would meet all of these criteria. In addition for many diseases there are no appropriate vaccines.

Generally, most research into vaccines has focused on the pathogens and the identification of pathogen-encoded vaccine candidates, and less on the host response. Yet, in most vaccine studies, evidence of variation in response has been reported, with varying proportions of non-responders, even after several immunisations, as well as individuals with adverse reactions. However this variation has mainly been ignored, or considered as inevitable animal-to-animal variation. On the other hand this suggests that at least some of the observed variation may be genetic and understanding what leads to non- or low-response, or pathology at the other extreme might identify new targets both for immunomodulators as well as for immunotherapeutics. These genes may also highlight new pathways that regulate the response to pathogens and be of more general importance. Genetic variability in response to vaccination is likely to become an even more significant factor in designing ideal “safe, synthetic” vaccines. The genes identified might also be important for disease resistance traits, and could potentially provide the tools to select “good responders”, as originally suggested by Wilkie and Mallard (1999) and more recently by Gay et al. (2007). However, the genes underlying variation in vaccine response have not been greatly explored in either humans (Poland et al., 2008) or livestock (Glass, 2004).

Probably the most complex issue in aims to identify genes controlling disease resistance or vaccine response relates to the choice of phenotype(s). Depending on the pathogen, the goal may be to prevent infection completely or more likely to reduce the consequences of infection, particularly those that impact on performance traits such as growth, milk and meat quality. Although it has been reported that selecting for performance has had a detrimental effect on resistance to infectious disease, for example milk yield and mastitis (Heringstad et al., 2005), the relationship between performance and disease resistance can show beneficial correlations. Susceptibility to *Mycobacterium bovis* is negatively genetically correlated with milk yield (Brotherstone et al., 2010) as well as survival (Bermingham et al., 2010) and susceptibility to *M. avium* subspecies *paratuberculosis* (MAP) is negatively correlated with several productivity traits including a combined productivity score, Net Merit (Attalla et al., 2010). Although these results need confirmation in other populations, they would suggest that selection for disease resistance and improved performance is a realistic goal. Furthermore the livestock industry routinely selects for a number of different criteria using a selection index of weighted traits (for example, Haile-Mariam et al., 2010). Thus, it should be possible to incorporate both disease resistance and performance as goals for selection. However, identifying relevant phenotypes for both infectious disease resistance and vaccine response is not straightforward, as in many cases the correlates of protection are unclear and/or difficult to measure.

We have undertaken a number of approaches to begin to address the hypothesis that small sequence changes (polymorphisms) in key host immune response genes, results in much greater changes in immune outcome. The first steps in trying to identify these genes is to determine if there is in fact any variation in phenotype – in this case in vaccine or immune responsiveness – and if so, to determine if any phenotypic variance can be accounted for by genetics. Usually this involves a large number of animals and linked phenotypes, in which the observed variation is partitioned into “environmental effects” (i.e. year of sampling, farm, etc.) and genetic effects (i.e. sire, line, breed, etc.) using multivariate statistical methods such as restricted maximum likelihood (REML) methods. The proportion of variance in the population being tested attributed to genetic factors is referred to as “heritability” or “*h*^2”^. In general, most disease resistance studies based on field studies report relatively low heritability e.g. mastitis studies typically report *h*^2^ ∼ 0.05 (Rupp and Boichard, 2003). However, these are likely to be underestimates as they are often based on treatment records or outcome at slaughter and are thus not very accurate. They are also based on all animals in a study and it is often unknown whether all the animals were exposed or not. This is particularly important as in many cases the resistance or tolerance phenotype is only expressed following exposure. Thus low estimates of heritability should not necessarily be viewed as a stumbling block to further investigation (Bishop and Woolliams, 2010). In addition heritability depends on the population and its environment, and does not describe the nature of the underlying genes, their number or impact on phenotype.

Generally, once variation has been established, two main types of study in which the phenotype is correlated with the genotype are undertaken. These involve linkage (in which the phenotypes are linked to markers inherited within family groups) or association (in which unrelated individuals are divided according to phenotype and the frequencies of markers within each group are compared). Investigation into the complexities of immune or vaccine responsiveness can take complementary approaches: either candidate genes previously determined by knowledge of mechanisms leading to protection or pathology, or whole genome approaches which do not rely on a priori discoveries and use markers distributed across the entire genome. In the past, association studies have mainly relied on candidate gene polymorphisms, whereas whole genome studies have employed linkage analysis with microsatellite markers to identify chromosomal regions that affect the phenotype: so called quantitative trait loci (QTL). Only a limited number of studies in livestock have identified QTL for disease resistance and even fewer for immune/vaccine response. Until recently, these have mainly involved a relatively limited number of microsatellite markers, and the chromosomal regions identified as having significant impact on the traits have been very broad and contained large numbers of candidate genes. However with the advent of livestock genome sequences and identification of very large numbers of single nucleotide polymorphisms (SNPs), it is now possible to conduct association analyses with thousands of SNPs as markers spread across genomes which should make it easier to identify causal genes (reviewed by Fan et al., 2010). For example, using the Illumina Bovine 50 Bead chip which contains approximately 50,000 SNPs, several studies have recently reported multiple loci controlling infection with or antibody response to MAP (Settles et al., 2009; Minozzi et al., 2010; Pant et al., 2010; Kirkpatrick et al., 2011). However, there is only limited concordance between the studies in terms of identified loci, some of which may relate to differences in MAP trait definitions. Higher density SNP arrays are now available for cattle containing over ten times the number of SNPs in the Illumina Bovine 50 Bead chip which in theory means that details of pedigree structures are probably no longer necessary, and should make genetic studies more tractable for livestock. Nonetheless although there has been considerable investment in the technology and resources for large-scale genotyping in cattle and other domestic species, the issues surrounding the “phenotype” gap remain to be solved.

Control of viral pathogens poses particular problems for livestock management because of their very nature. Their ability to proliferate, mutate and to modulate the host immune response might suggest that host species could not evolve counter-measures fast enough to become resistant, yet there is considerable evidence indicating that genes involved in host defence are extremely diverse both within and between species (Barreiro and Quintana-Murci, 2010). Evidence for host genetic variation exists for viral diseases in many host species including cattle (Glass et al., 2010) and the remainder of this article will focus on the genetics of bovine respiratory disease and vaccine responses in cattle.

## 2 Bovine respiratory disease

Bovine respiratory disease (BRD) is a major welfare and economic burden affecting both beef and dairy cattle, and costing around $750 million per annum in the United States (Snowder et al., 2006; Miles, 2009). BRD is a complex of diseases with many viral and bacterial infectious agents. The most common viral pathogens which are known to contribute to the development of BRD include bovine respiratory syncytial virus (BRSV), parainfluenza virus (PIV)-3, bovine herpes virus (BHV)-1 and bovine viral diarrhoea virus (BVDV) (Fulton, 2010). Often infection with viral respiratory pathogens results in secondary bacterial infections, including *Mannheimia haemolytica*, *Histophilus somni* and *Pasteurella multocida* (Griffin et al., 2010). Currently the control methods for BRD include good management, antibiotics and vaccination against the viral pathogens, BHV-1, BVDV, BRSV and PIV-3 (Bowland and Shewen, 2000) as well as vaccination against the bacterial pathogens, *M. haemolytica* and *P. multocida* associated with BRD (Fulton, 2010), although the efficacy of these vaccines has mainly not been subject to controlled challenge studies (Bowland and Shewen, 2000). The pathogens involved in BRD have co-evolved with their hosts and developed strategies for manipulating and evading the host immune response and this enables these pathogens to persist, and may be at least partly responsible for the failure of current vaccines to control BRD (Srikumaran et al., 2007).

A number of studies have indicated that different breeds of cattle have different degrees of susceptibility to BRD and heritability has been estimated to be around 0.04–0.08 (Muggli-Cockett et al., 1992; Snowder et al., 2005, 2006, 2007; Heringstad et al., 2007). It is possible that susceptibility to BRD is greater than estimated for the reasons described above, especially as the specific pathogens causing BRD are often unknown. In any case the genes underlying any genetic differences between animals or breeds are unknown.

## 3 Bovine respiratory syncytial virus

One of the major pathogens causing BRD as well as bovine shipping fever is considered to be BRSV which is a large enveloped, negative sense single stranded RNA *Pneumovirus* of the *Paramyxoviridae* family. It is ubiquitous in both dairy and beef cattle worldwide. Different strains of RSV also infect sheep, goats and humans. The clinical signs of BRSV include severe infection of the lower respiratory tract, resulting in coughing and nasal discharge and abnormal breathing sounds (Antonis et al., 2003). However, its effect on the host is variable from mild to severe; BRSV causes high morbidity in young animals and is the most important cause of lower respiratory tract infection in young calves (Valarcher and Taylor, 2007). By nine months of age over 70% of calves are estimated to have been infected with BRSV. Intensification of farming has probably increased the prevalence of BRSV in livestock. Thus this pathogen alone has high economic impact.

Currently the control of BRSV infection is mainly through management practices that reduce the level of circulating pathogen as well vaccination with modified live virus (MLV) or killed virus vaccines (Meyer et al., 2008). However, there is little concensus about which vaccines are the most efficacious as most studies conducted have been experimental trials and there are few field studies demonstrating clinical protection or reduction in clinical disease with any BRSV vaccine (Meyer et al., 2008). One of the most common MLV vaccines used in Europe is Rispoval RS (Pfizer) which is based on strain RB-94 (1969 Belgian isolate) (Zygraich, 1982).

Infection with BRSV, as with its human counterpart, HRSV, does not induce long lasting immunity (Meyer et al., 2008) and often calves are repeatedly infected before they become essentially immune to further infection. Similarly BRSV vaccines have limited protective capacity. Furthermore calves are particularly at risk as maternal antibody begins to wane. Yet, it is clear that this passively acquired antibody inhibits responses induced by vaccination (O’Neill et al., 2007; Meyer et al., 2008) and clearance of antibody is variable and at least in part genetically determined (O’Neill et al., 2006), making the timing of effective vaccination difficult to achieve. It is generally considered that neutralising antibody is the most important protective mechanism, and particularly mucosal antibody, but the precise role of different subclasses of immunoglobulin, in clearance of virus, maintenance of protection and induction of pathology is less clear (Meyer et al., 2008). In cattle, as in mice and humans, it appears that protection is associated with both a Th1 type response involving interferon-γ (IFN-γ) as well as the corresponding IgG subclass, IgG2 (Mapletoft et al., 2006), and a Th2 type response involving IgG1 (Kalina et al., 2005). BRSV infection induces bovine major histocompatibility complex (MHC) (BoLA) class II restricted CD4^+^ and BoLA class I restricted CD8^+^ T cells (Taylor et al., 1995; Gaddum et al., 1996; Fogg et al., 2001) and depletion of T cells in the respiratory tract leads to delayed BRSV clearance in calves (Taylor et al., 1995), suggesting that cell mediated immunity must also be important for protection in cattle.

Although BRSV is cytopathic *in vivo* and is associated with pathology involving innate and adaptive immune cells, *in vitro*, cell death does not necessarily occur and depends on the cell type infected (Valarcher and Taylor, 2007). Furthermore, pathology does not correlate with viral load (Gershwin, 2007). These findings suggest that the pathology seen in cattle is related more to the host immune response than to the virus (Gershwin, 2007; Ellis, 2009).

There are no licensed vaccines for human RSV, partly because formalin inactivated vaccines administered to babies, resulted in higher levels of pathology following natural infection with HRSV (Meyer et al., 2008). It has been suggested that these vaccines induced a non-protective Th2 biased response and that this was then invoked by natural infection and caused the observed pathology (Meyer et al., 2008). Similarly, experimental administration of inactivated BRSV vaccines can result in pathology following challenge with live BRSV, which appears to be mediated by an exacerbated Th2 mediated response including eosinophilia and IgE and suppressed IFN-γ (Th1) response (Gershwin, 2007). Nonetheless, these type of vaccines for cattle have been in use for many years and generally not found to result in such disease enhancement (Meyer et al., 2008). Thus it currently remains difficult to draw firm conclusions about the likelihood of inactivated BRSV vaccines inducing protective or pathogenic responses to natural BRSV infection. As with all BRSV vaccine responses, it seems likely that both vaccine and host factors such as strain of BRSV, dose, differences in adjuvants, maternal antibody and age, may account for some of the variation between animals and experimental studies. However, the role of host genetics in BRSV vaccine induced pathology and protection in cattle has up to now not been explored.

## 4 The role of genetics in determining the outcome of BRSV infections and vaccination

Although a role for genetics in variability in clinical outcome for RSV infection has been evinced in human studies (Janssen et al., 2007; Miyairi and DeVincenzo, 2008; Glass et al., 2010), such evidence is lacking for cattle and BRSV. However as the pathology in BRSV and HRSV shows many similarities, it seems likely that genetics must play a role (Glass et al., 2010). Furthermore, the evidence of genetics playing a role in BRD (see above) and the strong correlation of seropositivity for BRSV with BRD might also indicate a role for genetics in the bovine response to natural infection with BRSV. In addition, variation in the clearance of circulating maternal antibody was also shown to have a genetic component (O’Neill et al., 2006).

In contrast to the genetics of natural infection with BRSV, the response to vaccination with a MLV BRSV vaccine (Rispoval RS) has been linked to genetics. BRSV-specific IgG1 and IgG2 levels were measured prior to and following vaccination of approximately 500 young calves, and found to be highly variable between animals. These parameters were chosen as potential indicators of Th2 and Th1 responses in cattle respectively (Estes and Brown, 2002) as it has been hypothesised that genetic control of these pathways may underlie susceptibility and resistance to many pathogens. Furthermore both isotypes appear to be important for protection against BRSV (Kalina et al., 2005; Mapletoft et al., 2006). Heritabilities for serum IgG1 and IgG2 responses to vaccination were estimated to be as high as 0.36 at day 35 following vaccination, and significant sire effects were also discovered (O’Neill et al., 2006). This study exploited an experimental second generation cross population of dairy (Holstein) and beef (Charolais) cattle for which pedigrees were established. This population was also genotyped for 165 microsatellite markers and also for BoLA class II DRB3 alleles by a modified sequence based typing method of the polymorphic second exon of DRB3 (Baxter et al., 2008). QTL studies on production related traits on this population have revealed many QTL (Gutierrez-Gil et al., 2007, 2008a,b, 2009, 2010), and in addition several immune-related studies have now been carried out. In addition to the BRSV study (O’Neill et al., 2006), a genetic component to the T cell response to a mastitis causing pathogen, *Staphylococcus aureus*, has been reported (Young et al., 2005). Furthermore, following immunisation of this herd with a 40-mer peptide derived from foot-and-mouth disease virus (FMDV), a large number of QTL (Leach et al., 2010) and BoLA DRB3 polymorphisms (Baxter et al., 2009) have been associated with antibody responses to this peptide.

We have now found that, as with the response to the FMDV peptide (Leach et al., 2010), many regions of the bovine genome are strongly associated with the levels pre- and post-vaccination with the BRSV vaccine (summarised in Table 1). The variance in the phenotypic traits (total BRSV specific IgG, BRSV specific IgG1 and BRSV specific IgG2) explained by each QTL was around 2–3%. It can be seen that some chromosomal regions (on BTA 10 and 15) only influenced the clearance of maternally derived antibody and not the response to vaccination whereas others (on BTA 7, 8, 9, 14, 18, 23 and 24) only influenced the response to vaccination and not the level of antibody prior to vaccination. Only two chromosomes (BTA 2 and 17) harboured genes that influenced the level of antibody both pre- and post-vaccination. Interestingly, the level of variation of IgG2 at 14 days post vaccination appears to be the most polygenic trait, with six different chromosomal regions significantly associated with this trait, in contrast to the variation in the IgG1 levels at the same time point, where only a single QTL was detected. Since IgG1 and IgG2 are believed to be controlled by Th2 and Th1 cells, respectively (Estes and Brown, 2002), these results suggest that these pathways are influenced by different genes. It is also apparent that the early and later responses to vaccination are controlled by different genes. It is worth noting that the QTL on BTA23 whilst they have broad confidence intervals, do cover the bovine MHC region suggesting that the bovine MHC may be involved in determining the level of antibody response to the BRSV vaccine (and see below).

In almost every immune-related study for which there is evidence for a genetic component, the MHC has been identified as playing a role (Glass, 2004). Given the likely important role for cell mediated immunity in BRSV infection, including CD4^+^ and CD8^+^ Tells (Taylor et al., 1995), it would seem likely that polymorphisms in MHC genes would affect both the outcome from infection and also vaccination. However, although many candidate genes have been associated with outcome of HRSV infection in human studies, none have reported MHC associations for human or bovine RSV infection or vaccination (Janssen et al., 2007; Miyairi and DeVincenzo, 2008; Glass et al., 2010), although a very recent study in congenic strains of mice has shown that MHC haplotypes play a role in the susceptibility of neonatal mice (Tregoning et al., 2010). We have now discovered a number of significant associations between BoLA class II DRB3 polymorphisms and both the circulating level of antibody prior to vaccination as well as the antibody response to the BRSV vaccine (Tables 2 and 3). Although a few DRB3 alleles were significantly associated with both pre-vaccination and post-vaccination levels of anti-BRSV antibody (Table 2), there were very few animals in the herd that carried these alleles and the results must be treated with caution, and require verification in other populations. However, the majority of polymorphisms found in MHC class II DRB genes are concentrated in exon 2 and particularly in the sequences encoding the peptide binding pockets. These are the major binding anchors for presented peptides. Thus BoLA DRB3 alleles can be grouped into a series of peptide binding pocket motifs (Sharif et al., 2000; Baxter et al., 2009). Several individual positions within three of these pockets were significantly associated with the pre- and post-vaccination antibody levels to BRSV (Table 3). It is striking that none of the associations were significant for both the pre- and post-vaccination BRSV specific antibody levels. Perhaps not surprisingly this suggests that the rate of clearance of maternal antibody is controlled by different mechanisms from those operating in the induction of an adaptive response. The relative paucity of significant associations reported here are in marked contrast to those found with the FMDV peptide (Baxter et al., 2009) where several alleles and pocket positions were highly significantly associated with response. There are likely to be many reasons for this, not least that the FMDV 40-mer peptide has a limited number of epitopes whereas BRSV is likely to have many different epitopes recognised by the host immune system. In addition, the BRSV antibody levels are confounded with the pre-vaccination levels of circulating antibody. Nonetheless it is interesting that the pocket with the greatest associations was pocket 4 and this mirrors the findings with the FMDV peptide response (Baxter et al., 2009). Pocket 4 is located centrally within the peptide binding groove of DR molecules and has been implicated as playing a significant role in human immune responsiveness (Ou et al., 1998). This might indicate that in terms of vaccine design for any pathogen, ensuring that the vaccine includes epitopes that bind strongly to the major pocket 4 variants of DRB3 would ensure that all animals within herds would be protected.

Of course in terms of candidate genes for the vaccine response to BRSV, it is unlikely that only the MHC region would be important. As we (Clapperton et al., 2009; Glass et al., 2010) have argued elsewhere, genes expressed in the innate immune system are strong candidate genes for infectious disease associated traits in livestock as (a) activation of the innate immune system is essential and indeed determines the nature of the acquired immune response, (b) the genes involved are present in the germ-line, are highly polymorphic and code for molecules involved in the initial interactions of pathogens and the host defence system and (c) these molecules include pattern recognition receptors and soluble molecules (PRR and PRM) that sense a wide range of pathogens. PRMs and PRRs interact with pathogen associated molecular patterns (PAMPs) which are conserved on pathogens but generally not found in host species. The most well known PRRs are the Toll-like receptors (TLRs), but more recently a number of other PRR families have been described including the RNA helicase-type retinoic acid-inducible gene-1 receptors (RIG-1-like helicases) and NOD-like receptors which appear to be relevant for host detection of viruses (reviewed by Brennan and Bowie, 2010). Many candidate gene variants have been associated with severe humans RSV infections including PRMs – collectins, surfactant proteins A–D and mannose binding lectin (MBL), and the PRRs – TLR4 and TLR8 (Janssen et al., 2007; Miyairi and DeVincenzo, 2008; Glass et al., 2010). Although TLR2, TLR3 and RIG-1 have all been implicated in host interactions with RSV, no genetic associations in human studies have been reported. In cattle, a number of distinct collectin genes form a cluster on BTA28 (Gjerstorff et al., 2004), and some have been associated with viral pathogen interactions (Lillie et al., 2005). Polymorphisms have been described in bovine MBL (Wang et al., 2011) and bovine conglutinin levels are heritable (Holmskov et al., 1998). Similarly, bovine viral recognition *TLR*s (Cargill and Womack, 2007), and *NOD2* (also known as *CARD15*) harbour polymorphisms (Taylor et al., 2006) and a single SNP has been reported in bovine *RIG-1* (Cargill et al., 2006). Some associations of TLR variants with bacterial but not viral diseases have been reported in cattle. We have argued that TLRs and their intracellular down-stream signalling components may be the most likely candidates for many infectious disease traits in livestock species (Jann et al., 2009; Werling et al., 2009). Furthermore, as adjuvants are essential to invoke responses to vaccine components by triggering innate immunity, we suggest that gene variants encoding innate immune system proteins may also influence vaccine responsiveness. Although many SNPs in candidate genes have been proposed, very few studies in livestock have reported significant associations and this may in some cases be because the SNPs have no functional consequences. Determining whether individual SNPs are functionally relevant requires complex molecular biology and we have proposed that a simpler approach to narrow down the candidates is to consider only those SNPs that are both non-synonymous and positively selected (Jann et al., 2008). Of course this excludes SNPs that are non-coding and we accept that it is likely that SNPs in promoter regions can change expression levels and therefore could also have functional consequences. However even in human populations the relative importance of coding SNPs and non-coding SNPs in determining complex disease is as yet unknown, although it is clear that there is considerable inter-individual variation in gene expression across the human genome (Cookson et al., 2009; Skelly et al., 2009). With the advent of massive parallel sequencing for transcriptomics the contribution of inherited variation in gene expression to complex traits should become clearer (Majewski and Pastinen, 2011).

However we decided to test whether our proposal has merit and investigated whether any non-synonymous and positively selected SNPs in bovine TLRs were associated with the response to the BRSV vaccine and also to the FMDV peptide. In addition, the maximum number of microsatellite markers per chromosome in the QTL study was six, and thus as discussed above large confidence intervals were obtained which contain hundreds of candidate genes. Clearly this limits both the power and accuracy of the study, and in order to narrow the regions, we added additional SNP markers within some of the QTL. We found that a number of SNPs on different chromosomes were significantly associated with the response to the BRSV vaccine (Table 4) and also to the FMDV peptide (results not shown). The variance of the phenotypic trait accounted for by individual SNPs was in general much higher than for the QTL study, with some explaining over 10% of the phenotypic variance. However the majority of SNPs were not on the same chromosomes as the QTL, and this may well reflect the size of the study (∼500 animals) and the fact that the phenotypes were measured on a F2 generation which means that little recombination will have occurred. However as with the QTL study it is clear that both time and isotype of antibody must be under different genetic control. Although we tested for association of bovine SNPs in all ten TLRs, we only found two associated with response to the BRSV vaccine. Of particular interest, one of the most significant SNPs was on BTA 8, which influenced the IgG1 and IgG2 response to the BRSV vaccine and was located within the TLR4 gene. Further analysis determined that this SNP was non-synonymous and positively selected. Two further highly significant non-synonymous and positively selected SNPs were located in TLR8 and influenced the response to both BRSV vaccine and also the FMDV peptide. It is possible that both TLR4 and TLR8 are involved in the recognition of BRSV as there is evidence that HRSV F protein binds to TLR4 (Kurt-Jones et al., 2000) and BRSV induces a TLR4-dependent NFkB response in bovine TLR transfected cells (Lizundia et al., 2008). In addition TLR8 senses ssRNA and RSV is an RNA virus although no direct interactions between TLR8 and RSV have been reported. In humans polymorphisms in TLR4 and TLR8 have been associated with severity of BRSV infection (Janssen et al., 2007).

## 5 Concluding remarks

**Fig. 1:**
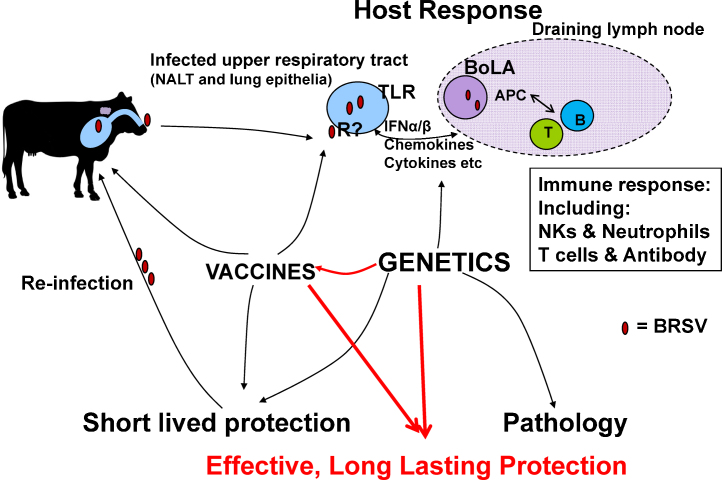
Schematic diagram illustrating the potential role of genetics in the variation in outcome following natural infection or vaccination with BRSV. Understanding the relationship that genetics has with outcome may lead to improvements in host disease resistance and/or vaccine efficacy.

In conclusion, genetics clearly influences both the response to vaccination and also to infection. In addition, genetics also plays a key role in the rate of clearance of maternal antibody. Although we have identified a number of candidate variants, which include BoLA DRB3 and TLRs 4 and 8 as playing a significant role, we have also identified many regions that may harbour novel genes that are important in determining the response to vaccination. Because of the strong linkage between genes in the BoLA region, we currently cannot disentangle whether our data supports the role of BoLA DRB3 or is linked to polymorphisms in other classical class II genes (DQA and/or DQB) or indeed class I genes. However, the pocket 4 associations would strongly suggest that BoLA DRB3 is playing an important role in the observed variation in antibody response. We and others have demonstrated the importance and complexity of DQ molecules in T cell responses in cattle (Glass et al., 2000; Norimine and Brown, 2005; Gerner et al., 2009), and further exploration of the role of DQ in bovine responses to BRSV is warranted. We found three non-synonymous, positively selected SNPs in two TLRs providing some support to our hypotheses relating to the involvement of innate immune genes in disease resistance and vaccine responsiveness. The lack of evidence for association with SNPs in other candidate genes does not of course rule out a role for them, as it could simply be that the study was too small to detect their influence and/or other polymorphisms may not be represented in the study herd. Although the uncertainty surrounding the correlates of protection for BRSV remains to be solved, our initial studies suggest that another significant factor in observed variation in protection and pathology following natural infection and vaccination in cattle may be host genetics. However, in order to investigate further, the QTL and SNPs require validation in new and larger populations as well as more detailed molecular studies, for example cells transfected with different TLR variants may reveal the underlying mechanisms. Future studies that improve our understanding of the role of genetics in immune responsiveness and disease resistance have the potential to improve vaccine efficacy as well as provide selectable markers that breeders could use to improve livestock disease resistance as well as vaccine responsiveness (Fig. 1).

## Conflict of interest

None.

## Figures and Tables

**Table 1:** The sequential influence of significant quantitative trait loci (QTL) associated with BRSV specific antibody levels pre- and post-vaccination across time.

Day^a^/Trait^b^	Chromosome number (BTA^c^)											
	3	17	2	10	15	8	18	7	9	23	24	14
Pre-vaccination												
−14	X^d^	XX	X									
0: Total^e^ IgG			X									
0: IgG1			X		X							
0: IgG2				X								
Post-vaccination												
0–14			X			X	X					
14: Total IgG		X						X				
I4: IgG1		X										
14: IgG2	X						X		X	X	X	X
0–49			X									
49: Total IgG						X	X					X
49: IgG1							X					
Overall^f^ IgG1						X						X
Overall IgG2										X		

a Day is relative to vaccination at day 0.

b Trait = BRSV specific antibody level, measured by ELISA as detailed in (O’Neill et al., 2006).

c BTA = *Bos taurus* autosome. The order shown attempts to reflect the influence of different QTL across the time course, with those loci which influence the response at the earliest time point being shown first.

d Each X represents the presence of a significant QTL (*p* < 0.05); XX represents two QTL on the same chromosome.

e Total refers to IgG1 and IgG2 levels.

f Overall refers to the total amount of IgG isotype generated across time (calculated as “area under the curve” by the Trapezoidal rule). All QTL are at least 5% chromosome wide significant

**Table 2:** Significant *BoLA DRB3* alleles associated with BRSV-specific IgG levels pre- and post-vaccination.

DRB3 allele^a^	Pre-vaccination	Post-vaccination
*0801	<0.05^b^	N.S.
*0901	N.S.^c^	<0.05
*1002	N.S.	<0.05
*1701	N.S.	<0.05

a DRB3 alleles determined by a sequence based typing method of the 2nd exon of BoLA DRB3, as described in Baxter et al. (2008).

b *p* values determined by Wald test.

c N.S. = not significant.

**Table 3:** Peptide binding pockets significantly associated with BRSV-specific IgG levels pre- and post-vaccination.

DRB3 Pocket^a^	Pocket position	Pre-vaccination	Post-vaccination
4	β13	<0.05^b^	N.S.^c^
4	β70	N.S.	<0.05
4	β74	N.S.	<0.001
7	β28	N.S.	<0.05
7	β30	N.S.	<0.05
9	β37	<0.05	N.S.

a DRB3 Pockets determined as described in Baxter et al. (2009).

b *p* values determined by Wald test.

c N.S. = not significant.

**Table 4:** The sequential influence of significant SNPs associated with BRSV specific antibody levels at vaccination day and post-vaccination.

Trait^a^	Day^b^	Chromosome number (BTA^c^)												
		12	16	22	24	6	7	19	25	X	21	27	29	8
IgG2	0	X^d^	X	X	X									
	14					X	X							
	35							X	X	X				
	49								X		X			

IgG1	14			X					X			X	X	
	35						X							X
	49													

Overall^e^ IgG1												X		
Overall IgG2														X

a Trait = BRSV specific antibody level, measured by ELISA as detailed in (O’Neill et al., 2006).

b Day is relative to vaccination at day 0.

c BTA = *Bos taurus* autosome. The order shown attempts to reflect the sequential influence of different SNP across the time course, with those loci which influence the response at the earliest time point being shown first.

d Each X represents the presence of a significant SNP (*p* < 0.05).

e Overall refers to the total amount of IgG isotype generated across time (calculated as “area under the curve” by the Trapezoidal rule).
